# Design and development of primers for detection of Streptococcus pneumoniae, Haemophilus influenzae, and Neisseria meningitidis

**DOI:** 10.3205/dgkh000545

**Published:** 2025-04-30

**Authors:** Leila Azimi, Fatemeh Shirkavand, Shahnaz Armin, Fereshteh Karbasian, Hannan Khodaei

**Affiliations:** 1 Pediatric Infections Research Center, Research Institute for Children’s Health, Shahid Beheshti University of Medical Sciences, Tehran, Iran; 2 Department of pediatric gastroenterology and hepatology, Ali-Asghar children’s hospital, Iran University of Medical Sciences, Tehran, Iran

**Keywords:** Streptococcus pneumoniae, Haemophilus influenzae, Neisseria meningitidis, primer, sensitivity

## Abstract

**Background:**

The mortality rate of meningitis is still alarmingly high in certain regions across the globe. The objective of this research is to identify the most effective primers for detecting *Streptococcus (S.) pneumoniae, Haemophilus (H.) influenzae, *and* Neisseria (N.) meningitidis* using Real-Time PCR technology.

**Materials and methods:**

Two sets of primers were developed for detecting *S. pneumoniae, H. influenzae*, and *N. meningitidis* using the Primer Biosoft Allele ID 7.6 application. The study examined the minimum bacterial copy numbers detectable by each primer, as well as their specificity.

**Results:**

*CtrA* and *hpd2* could detect the 400 copy numbers/ml of *H. influenzae*, and *N. meningitidis* and *LytA2* could detect the 40 copy numbers/ml of *S. pneumoniae*. The sensitivity and specificity of all primers was 100% (CI: 95%).

**Conclusion:**

Using more sensitive primers to detect the bacterial agent responsible for causing bacterial meningitis increases the chance of identifying the causative bacteria. The primers designed in this study could identify the selected bacteria with at least 10 times more sensitivity than the currently available commercial diagnostic kits in Iran.

## Zusammenfassung

**Hintergrund:**

Die Sterblichkeitsrate bei Meningitis ist in bestimmten Regionen der Welt immer noch alarmierend hoch. Ziel der Arbeit ist es, die wirksamsten Primer für den Nachweis von *Streptococcus (S.) pneumoniae, Haemophilus (H.) influenzae *und* Neisseria (N.) meningitidis* mithilfe der Real-Time-PCR-Technologie zu ermitteln.

**Material und Methoden:**

Es wurden zwei Primer-Sets zum Nachweis von *S. pneumoniae, H. influenzae *und* N. meningitidis* unter Verwendung der Primer Biosoft Allele ID 7.6 entwickelt. Untersucht wurden die minimalen bakteriellen Kopienzahlen, die von jedem Primer nachgewiesen werden können, sowie ihre Spezifität.

**Ergebnisse:**

*CtrA *und* hpd2* konnten 400 Kopienzahlen/ml von *H. influenzae* und *N. meningitidis* und LytA2 40 Kopienzahlen/ml von *S. pneumoniae* nachweisen. Die Sensitivität und Spezifität aller Primer betrug 100 % (CI: 95 %).

**Schlussfolgerung:**

Die Verwendung empfindlicherer Primer zum Nachweis des für die bakterielle Meningitis verantwortlichen Erregers erhöht die Chance, die verursachenden Bakterien zu identifizieren. Die entwickelten Primer konnten die ausgewählten Bakterien mit mindestens zehnmal höherer Empfindlichkeit identifizieren als kommerzielle Diagnosekits im Iran.

## Introduction

*H. influenzae, N. meningitidis, *and* S. pneumoniae* can cause infection in important tissues such as blood and cerebrospinal fluid (CSF), requiring rapid intervention and healing [1], [2], [3], [4], [5]. Fast and early diagnosis of these bacteria is necessary for quick treatment and appropriate antibiotic therapy. Blood and CSF cultures (conventional culture or BACTEC) are traditional methods used to identify bacteria [6]. However, these methods are time-consuming and, in some cases, bacteria cannot grow in the culture medium due to the use of antibiotics and despite the clinical symptoms, bacterial growth is not observed. Molecular methods can be helpful in overcoming these disadvantages. 

Real-time PCR, a molecular method, can be used for early diagnosis and fast treatment of the patient. Many commercial diagnosis kits with different levels of sensitivity are used worldwide to identify the cause of infections. The sensitivity and minimum detectable copy numbers of bacteria are related to the primers of the kits. As such, the primers play a key role in these diagnosis kits. In this regard, the aim of this study was to design a diagnostic panel using real-time PCR to identify *H. influenzae, N. meningitidis, *and* S. pneumoniae*. 

## Materials and methods

### Samples and setting 

*H. influenzae, N. meningitidis, *and* S. pneumoniae*, which were isolated from bacterial meningitides and had their identity confirmed by real-time PCR in the last study by our group [7], were selected. These strains were used to evaluate the designed primers. 

### Primer design 

Two different pairs of primers were designed by Allele ID 7.6 for different genes or different parts of genes to detect *H. influenzae (hpd* gene), *N. meningitidis* *(CtrA* and *SodC*), and *S. pneumoniae* (*lytA*) (Table 1 (Tab. 1)). 

### DNA extraction

Bacterial genome extraction was performed using a DNA extraction kit (Qiagen Cat No./ID: 51304). The DNA concentration was read by Qubit. 10 dilutions were prepared by a factor of 101 for each bacterium, and the DNA concentration of all dilutions was determined by the Qbit instrument to determine the sensitivity and the cut-off point of these primers.

### Real-time PCR assay 

Real time-PCR assay was performed for the identification of bacteria using differently designed primers. Cyber green master mix and ABI step-one, in addition to the real-time PCR instrument, were used in this setup. 

The DNA copy number calculator works according to the following equation:


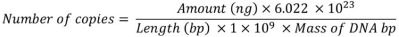


where Amount (ng) is the amount of DNA in nanograms (ng) in the tube, 6.022x1.023 is Avogadro’s constant and represents the number of molecules per mole, Length (bp) is the length of DNA, in base pairs (bp), in the template (*H. influenzae* 1.830.137 bp, *N. meningitidis* 2.184.406 bp and *S. pneumoniae* 2.160.837 bp), 1x10^9^ is the factor used to convert to ng, Mass of DNA bp stands for the average mass of a DNA bp, which is either 660 (dsDNA) or 330 (ssDNA) g/mole. This value depends on what is selected as the type of DNA in the calculator (https://toptipbio.com/dna-copy-number-calculator/).

The genomes of *Acinetobacter baumannii, Pseudomonsa (P.) aeruginosa, Escherichia (E.) coli, Klebsiella (K.) pneumoniae, Enterobacter *spp*., Staphylococcus (S.) aureus *and* Enterococcus *spp. were used as a negative control to evaluate the specificity of these primers. 

## Results

The results of real-time PCR showing the sensitivity of these primers to detect the included bacteria are presented in Table 2 (Tab. 2).

None of the duplication observed with the genomes of *A. baumannii, P. aeruginosa, E. coli, K. pneumoniae, Enterobacter *spp*., S. aureus *and* Enterococcus* spp. confirmed the 100% (CI: 95%) specificity of these primers. 

## Discussion

Bacterial meningitis occurs most often in childhood and the etiological pathogens can be diverse in different age groups of children [4], [8], [9]. Based on previous studies, the incidence of bacterial meningitis can vary depending on factors such as time, geographical location, and patient age [4], [8], [9]. 

A systematic review and meta-analysis on the worldwide etiology of bacterial meningitis showed that the most prevalent causative pathogens were *N. meningitidis *and* S. pneumoniae* in all age groups, while *S. pneumoniae* was most prevalent in children [4]. An accurate method to identify these bacteria can be helpful in saving human lives and prevent them from becoming disabled in the future due to meningitis. The results of this study showed higher sensitivity of *CtrA* primer than *SodC* for the detection of *N. meningitidis*. On the other hand, different genome regions of *hpd* and *LytA* genes in *H. influenzae *and* S. pneumoniae*, respectively, were selected to design primers. The different results when various genes are selected to identify the bacteria, showing that the primer selected for nucleotide sequence between 777–903 has greater sensitivity for detecting *H. influenzae*. Also, primer design for nucleotide sequences between 350–424 is more sensitive for detecting *S. pneumoniae*.

In the study by Haddad-Boubaker et al. the results showed that Real-time PCR could detect up to 67.10^–4^ ng/µL DNA for *S. pneumoniae*, 38.10^–6^ ng/µL and 38.10^–3^ ng/µL for *N. meningitidis* *ctrA* gene and *sodC* gene, respectively, and 97.10^–4^ ng/µL for *H. influenzae* [10]. In the current study, the primers used were *ctrA*, *hpd.2*, and *LytA.2*. These results are near to ours.

Cyber green was used in the current study and is more affordable in comparison to using the probe of the Haddad-Boubaker et al. study [10]. One of the commercial molecular-diagnosis kits for the detection of these three bacteria is being used (Sacace™ NHS Meningitidis Real-TM) in Iran. The analytical sensitivity, genome equivalents/ml of this kit is 1*10^3^ (genome of bacteria/ml) [11], but the primers we designed have a minimum detectable bacterial genome 4*10*^2^* for *N. meningitidis *and* H. influenzae* and 4*10 for *S. pneumoniae*. These results showed that the primers designed in the current study detected bacteria with at least 10 times greater sensitivity. This is an important advantage of molecular diagnosis kits, especially when patients used antibiotics before the test.

## Conclusions

The detection of the causative bacteria of meningitis can be helpful to choose the best therapeutic process as soon as possible. In this regard, selecting the most accurate and rapid method with the greatest possible sensitivity to identify relevant agents is necessary. It is important to know the exact cause of meningitis, because the choice of treatment depends on it.

## Notes

### Competing interests

The authors declare that they have no competing interests.

### Ethical approval 

Ethical approval No. IR.SBMU.RICH.REC.1399.061 was granted by the Pediatric Infections Research Center, Shahid Beheshti University of Medical Sciences, Tehran, Iran.

### Funding

The research reported in this publication was supported by the Researcher Grant Committee under grant number [20326] from the Pediatric Infections Research Center, Shahid Beheshti University of Medical Sciences, Tehran, Iran.

### Authors’ ORCID

- Azimi L: https://orcid.org/0000-0002-7216-2530
- Shirkavand F: https://orcid.org/0009-0001-5207-4883
- Armin S: https://orcid.org/0000-0002-4993-482X
- Karbasian F: https://orcid.org/0000-0003-2494-6231
- Khodaei H: https://orcid.org/0000-0002-2297-4733

## Figures and Tables

**Table 1:**
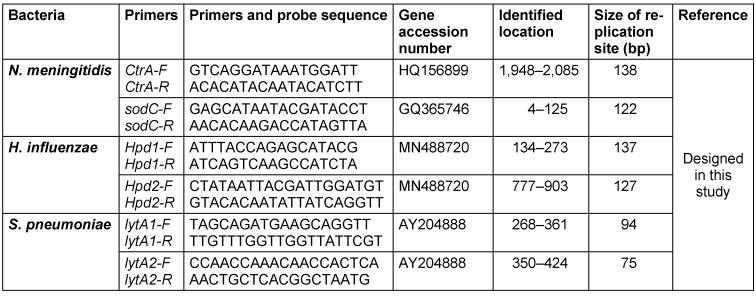
Primer sequencing of specific real-time PCR

**Table 2:**
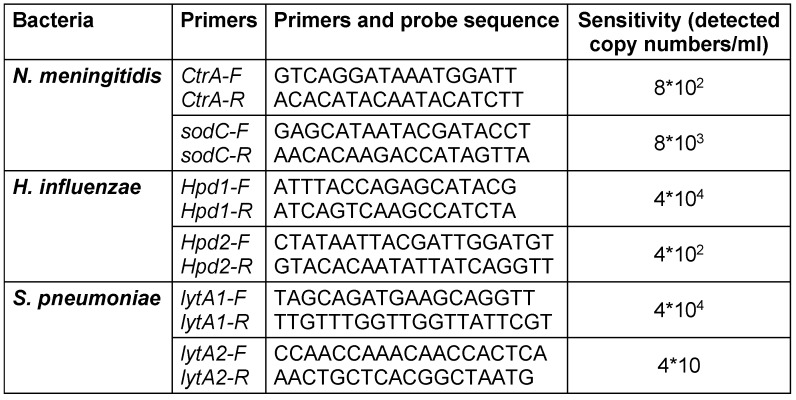
Sensitivity of designed primers
